# Self-related thought alterations associated with intrinsic brain dysfunction in mild cognitive impairment

**DOI:** 10.1038/s41598-025-97240-8

**Published:** 2025-04-10

**Authors:** Povilas Tarailis, Kim Lory, Paul G. Unschuld, Christoph M. Michel, Lucie Bréchet

**Affiliations:** 1 Functional Brain Mapping Laboratory, Department of Fundamental Neuroscience, University of Geneva, Geneva, Switzerland; 2 Geriatric Psychiatry Service University Hospitals of Geneva (HUG), Thônex, Switzerland; 3 Department of Psychiatry, University of Geneva, Geneva, Switzerland; 4 Center for Biomedical Imaging (CIBM), Lausanne, Switzerland; 5 Department of Clinical Neurosciences, University of Geneva, Campus Biotech Chemin des Mines 9, Geneva, 1202 Switzerland

**Keywords:** Healthy aging, Cognitive decline, MCI patients, Resting-state EEG microstates, Self-related thought, Cognitive ageing, Predictive markers

## Abstract

The subjective experience of self-awareness is attributed to the human capacity for introspective thought during periods of mind-wandering. However, how this cognitive function is impacted in individuals with mild cognitive impairment (MCI) still needs to be better understood. To address this gap, we investigated alterations in self-referential thinking in a cohort of 30 MCI patients, comparing them to 60 healthy old-aged and 60 healthy younger controls. MCI patients exhibited a notable decline in overall cognitive function, as evidenced by significantly lower scores on the Montreal Cognitive Assessment (MoCA), with particular deficits in Memory subscore and Memory Index Score (MIS). Employing the Amsterdam Resting-State Questionnaire (ARSQ) to assess mind-wandering, we observed diminished self-related thoughts relating to personal past experiences and future thinking among MCI patients. Notably, using high-density electroencephalography (hdEEG) microstate analysis, we detected reduced neural activity for microstate C associated with self-related thoughts in MCI patients and healthy older relative to healthy younger controls, and an increase in neural activity for microstate A in MCI patients compared to healthy older and younger controls. This aberrant temporal activity was localized within brain regions implicated in episodic autobiographical memory and the default mode network. Our results highlight a link between impaired mind-wandering ability and dysfunction within the intrinsic neural networks of MCI patients, underscoring its implications for disruptions in the sense of self within this clinical population.

**Supplementary Information:**

The online version contains supplementary material available at 10.1038/s41598-025-97240-8.

## Introduction

We have all experienced moments when we encountered childhood friends but struggled to recall their names or entered a room only to forget what we planned to do there. However, when do these seemingly small episodes indicate an early stage of memory or cognitive ability loss, such as seen in mild cognitive impairment (MCI)? A fundamental characteristic of self-conscious experiences (autonoetic consciousness) is the ability to remember the past and predict the future^1^. This human capability to mentally travel to the past or future relies on episodic autobiographical memory^2^ and episodic future thinking^3^. Recently, we have examined an embodied component of self-consciousness (bodily self-consciousness) and demonstrated its relevance to episodic autobiographical memory^4–6^. Already 20 years ago, Addis et al.^7^ showed that impairments of self-identification, a core component of bodily self-consciousness, were associated with deficits in episodic autobiographical memory of both healthy older individuals and patients with Alzheimer’s disease.

It is well known that self-related episodic memories and episodic future thinking are impaired in patients with mild cognitive impairment (MCI)^8–11^. Over the past decade, cognitive neuroscience research revealed that the brain regions commonly engaged by episodic past and future thinking are part of the brain’s default mode network (DMN)^12,13^. This intrinsically oriented network becomes active during self-referential mind-wandering at rest^14–16^ when individuals think of themselves, remember their past, or plan their future^17^. Self-related, intrinsic, and conscious thoughts dynamically evolve from one moment to another, even in the absence of external stimuli, and commonly engage a set of brain regions, including the ventral medial prefrontal cortex, posterior cingulate cortex, inferior parietal lobule, lateral temporal cortex, dorsal medial temporal cortex, and the hippocampus. However, investigating the conscious experiences of the self during rest is challenging, as they are introspection-dependent^18–20^. Despite these challenges, the evaluation of self-related thoughts is relevant for the early detection of cognitive decline and memory loss, such as in dementia^21–23^.

Traditionally, the Montreal Cognitive Assessment (MoCA) serves as a widely used cognitive screening tool to evaluate global cognitive functioning and cognitive decline, particularly sensitive to MCI detection^24^. According to the International Classification of Diseases by the World Health Organization^25^, MCI is characterized by general decline from the individual’s previous level of functioning and mild impairment in one or more cognitive domains. Cognitive impairment is not attributable to normal aging and may be static, progressive, or may resolve or improve depending on underlying cause or treatment. However, diagnosis of neurodegenerative diseases, such as Alzheimer’s disease, based on the symptoms is not optimal since symptoms occur too late in the disease progression^26^. Thus, early diagnosis and development of potential biomarkers of memory and cognitive decline are currently some of the leading topics in the clinical and cognitive neurosciences^27,28^.

The electroencephalogram (EEG) records brain electrical activity, primarily generated by synchronous post-synaptic potentials of pyramidal neurons^29,30^. This non-invasive method thus enables the direct assessment of synaptic dysfunctions caused by neurodegenerative diseases, such as Alzheimer’s disease^31,32^. Various quantitative EEG analyses in frequency, time, and space domains are employed to study brain alternations in Alzheimer’s disease^33,34^. Among these, the broadband multichannel approach known as the EEG microstates has demonstrated promising results in the early detection of neurodegenerative diseases, such as Alzheimer’s disease^34–38^, and was suggested as the most robust marker to study cognitive decline^39^.

The microstate approach considers information from all channels simultaneously, thus capturing both temporal and spatial dynamics of large-scale brain electrical activity. This approach has been shown to identify a few highly reproducible topographies that explain a significant portion of data variability. Emerging literature over the last decade has demonstrated that the EEG microstate method is a powerful tool for assessing cognitive functions in healthy and altered brain states^40–44^. High-density EEG (hdEEG) combined with EEG source imaging can identify large-scale brain networks of synchronized activity during sub-second periods^45–47^. Building upon our previous hdEEG results^46^ and recent fMRI study^48^ showing altered thoughts associated with brain changes in the hippocampus, default, and frontoparietal networks, we predicted to find here supporting evidence for key regions implicated in internal mentation in MCI patients and healthy aging population using hdEEG microstate and source imaging techniques.

To evaluate mind-wandering in more detail, researchers recently developed a retrospective self-report questionnaire (the Amsterdam Resting-State Questionnaire, ARSQ)^49^. Interestingly, they found a significant age-related effect on self-related aspects of mind-wandering: “Self,” pertaining to self-related thoughts; “Planning,” involving future-directed thoughts; and “Visual Thoughts,” focusing on what-where imagery during mind-wandering. Several studies reported the relevance of assessing spontaneous thoughts during rest to understand the intrinsic brain activity reflected in the EEG microstates^50–52^. To our knowledge, this resting-state questionnaire has not yet measured the self-related conscious thoughts of MCI patients.

In the present study, we used hdEEG to investigate spontaneous mentation in 30 MCI patients, 60 healthy younger and 60 older participants. Our primary aim was to identify self-related changes in the mind-wandering patterns of MCI patients assessed with cognitive questionnaires and brain electrical activity of EEG microstates. Building upon our prior research^53^, we expected a classical decline in cognitive functioning among MCI patients, as measured by the MoCA scores. Specifically, we expected a memory decline in MCI patients compared to healthy controls, captured by the MoCA Memory subscore and the Memory Index Score (MIS). Additionally, using the ARSQ, we expected alterations in the self-related thoughts of MCI patients and healthy older participants. Drawing from our previous findings regarding the functional significance of microstate C to self, consciousness, and memory^41,42,46,54^, we anticipated significantly lower temporal parameters of this self-related microstate in the MCI patients. Finally, we analyzed the underlying sources of EEG microstates and expected decreased activation of this specific EEG microstate, reflecting dysfunction within the self-related memory network and the default mode network among MCI individuals.

## Results

### Early detection of memory and cognitive decline in MCI patients

Here, to study the overall cognitive functioning and detection of cognitive changes in individuals with MCI compared to a healthy population, we first aimed to confirm that the overall MoCA scores differed among our MCI patients compared to healthy older and younger controls.

**Fig. 1:**
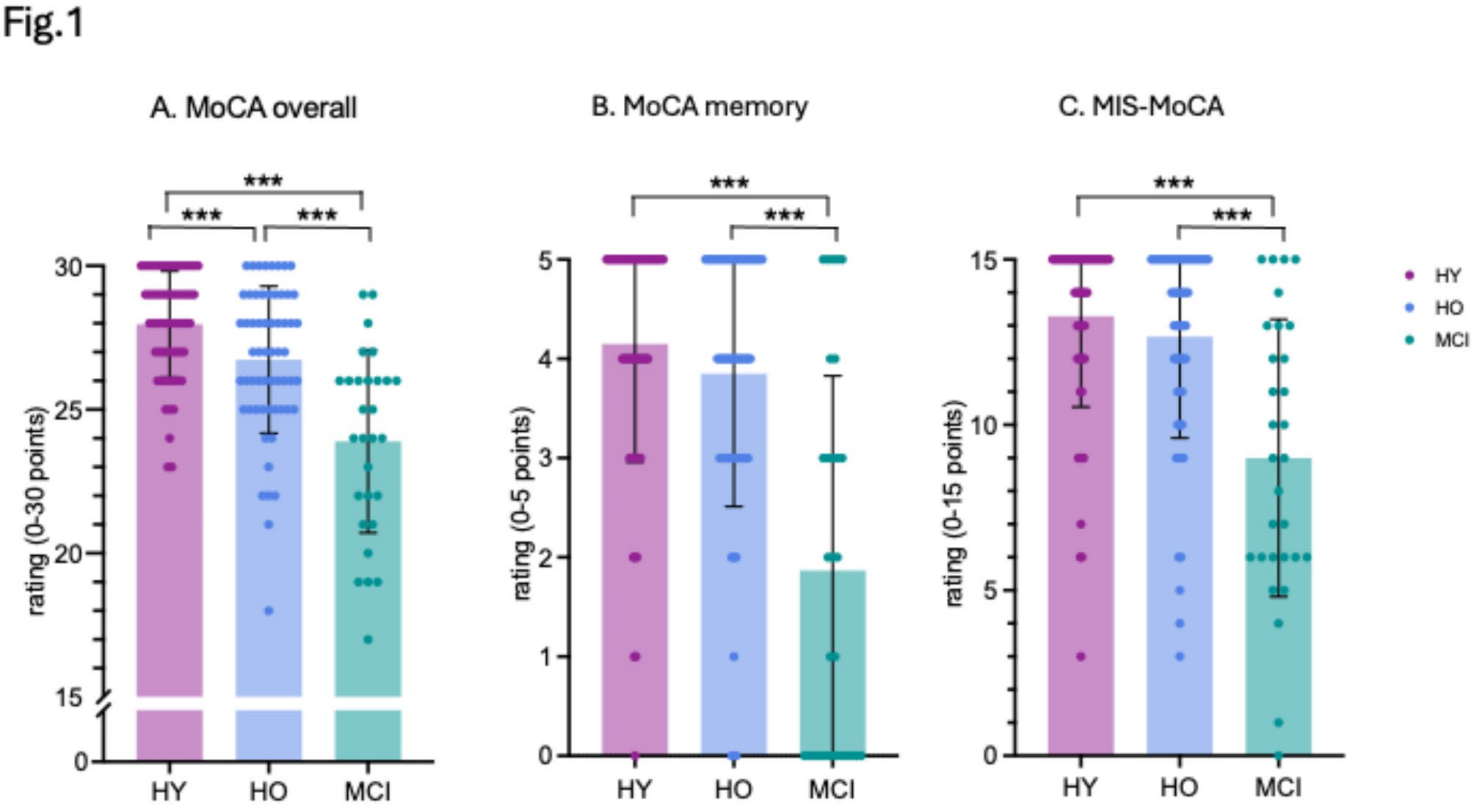
(**A**) MoCA scores of the overall, (**B**) memory subscore and (**C**) memory index score (MIS-MoCA). Error bars indicate standard deviations. P values are FDR corrected. *** *p* < 0.005.

We found a classical decline in overall MoCA performance, with significantly worse cognitive functioning of MCI patients than healthy older and younger participants (Fig. 1A) (MCIs: M = 23.40, SD = 4.15; HO: M = 26.73, SD = 2.56; HY: M = 27.97, SD = 1.85; F (2,147) = 28.41, *p* < 0.001). This finding confirms the decline in cognitive functioning among MCI patients compared to healthy older and younger participants. Interestingly, healthy older individuals also differed significantly from the younger participants in the overall MoCA score, indicating cognitive challenges already appearing but not reported in some participants.

Next, we explored potential differences in the memory subscore of MoCA and the Memory Index Score of MoCA (MIS-MoCA) in MCI patients compared to healthy controls. We hypothesized that individuals with MCI would perform worse than healthy older, and younger participants on these memory tasks. As anticipated, we found that MCI patients showed significantly lower memory subscore (Fig. 1B) (MCIs: M = 1.87, SD = 1.96; HO: M = 3.85, SD = 1.34; HY: M = 4.15, SD = 1.19; F (2,147) = 27.35, *p* < 0.001) and memory index score (Fig. 1C) (MCIs: M = 9.00, SD = 4.19; HO: M = 12.67, SD = 3.06; HY: M = 13.28, SD = 2.74; F (2,147) = 19.07, *p* < 0.001) compared to healthy older and younger participants. Detailed results of all MoCA subscores are provided in Supplementary Materials (SI Appendix, Table S1).

### Self-thoughts decline in MCI patients and healthy aging population during mind-wandering

The Amsterdam Resting-State Questionnaire (ARSQ) is a validated tool that efficiently explores self-related, conscious experiences during rest^49^. We have previously demonstrated the relevance of understanding spontaneous thoughts during rest to gain insights into the intrinsic brain activity of healthy young participants^51,55,56^. Here, we asked whether changes in self-related thoughts during mind-wandering episodes are visible in the MCI population compared to healthy controls. We hypothesized that self-related thoughts of the ARSQ, particularly within the Self, Planning, and Visual domains, would demonstrate a decline in healthy aging (similar to^49^) and pathological populations compared to healthy young participants.

**Fig. 2:**
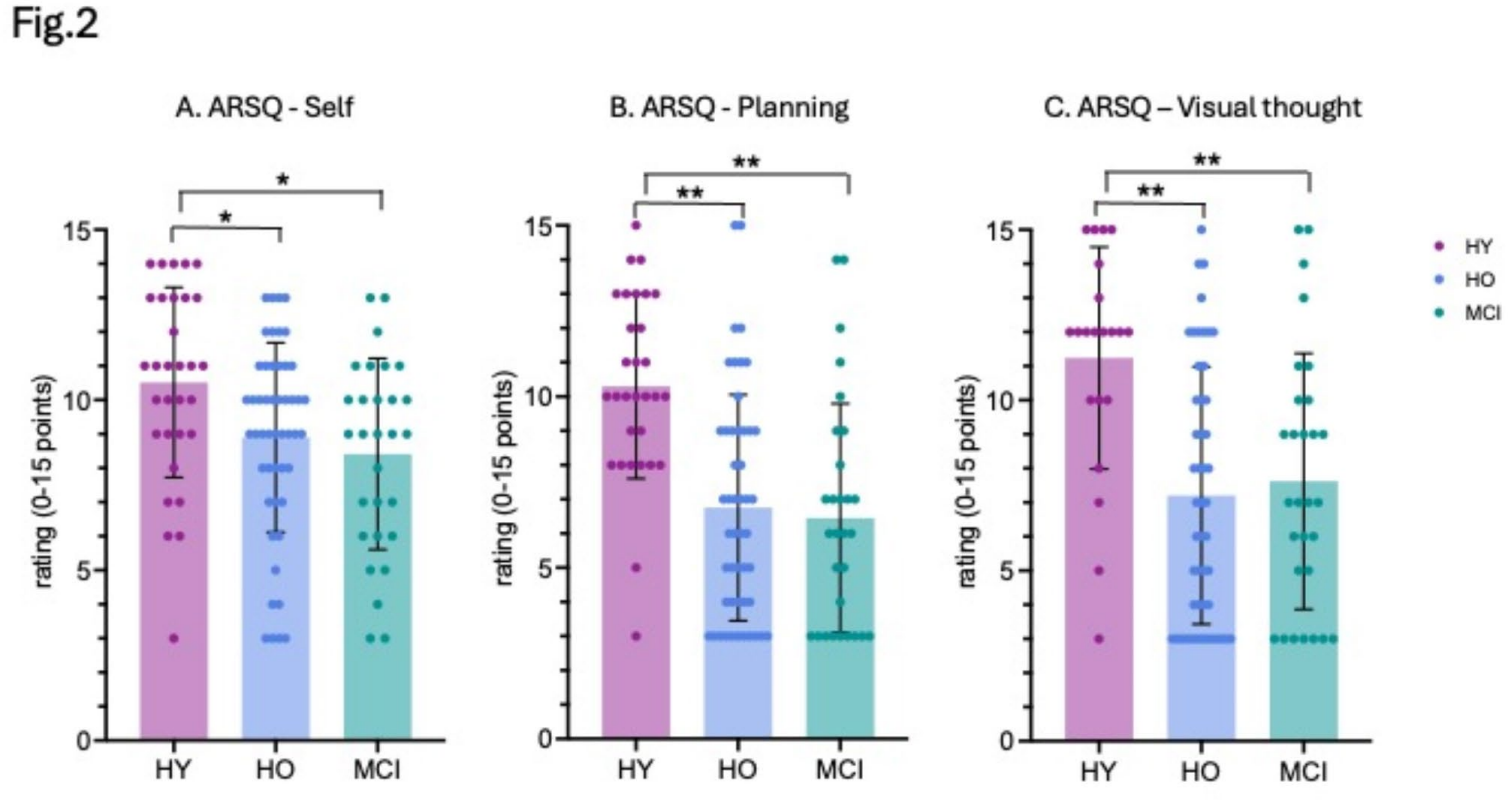
(**A**) ARSQ scores of self, (**B**). Planning, and (**C**) Visual thought. Error bars indicate standard deviations. P values are FDR corrected. *p < 0.05, **p < 0.005.

We found that the three self-related categories (Self, Planning, and Visual thoughts) assessed through the behavioral resting-state questionnaire decreased in both healthy older, and pathological populations. MCI patients had significantly fewer self-thoughts related to the past (M = 8.14, SD = 2.81) when compared with the healthy young participants (M = 10.50, SD = 2.81; *p* = 0.011, d = -0.729) and there was also a significant difference between healthy older (M = 8.92, SD = 2.81, *p* = 0.027, d = -0.556) and healthy younger participants (Fig. 2A). We also observed a significant decrease in self-thoughts related to the future in MCI patients (M = 6.45, SD = 3.34, *p* < 0.001, d = -1.254) and healthy older participants (M = 6.76, SD = 3.30, *p* < 0.001, d = -1.137) compared to healthy younger participants (M = 10.30, SD = 2.69) (Fig. 2B). Similarly, there was a significant decrease in visual thoughts with MCI patients (M = 7.62, SD = 3.76, *p* = 0.002, d = -1.001) and healthy older participants (M = 7.20, SD = 3.76, *p* < 0.001, d = -1.099) exhibiting fewer visual thoughts compared to healthy younger participants (M = 11.24, SD = 3.25) (Fig. 2C). While the ratings in these categories were lower in MCI participants compared to healthy older participants, these differences did not reach significance (self: *p* = 0.313; planning: *p* = 0.165; visual thought: *p* = 0.581). Detailed results for all ARSQ categories can be found in Supplementary material (SI Appendix, Table S2).

### “Self-experience” memory network showed decreased activity in MCI patients and healthy older participants

EEG microstate analysis allows the investigation of temporal dynamics of large-scale neural networks and provides information about the functional organization of spontaneous mind-wandering^40^. In line with our previous findings in healthy younger participants^41,46,54^, we assumed that the neural network underlying microstate C reflects the self-relevant memory processes. Here, we hypothesized that the global explained variance (GEV) and the temporal parameters (occurrence, coverage, and duration) of microstate C would exhibit decreased activity in MCI patients compared to healthy controls.

**Fig. 3:**
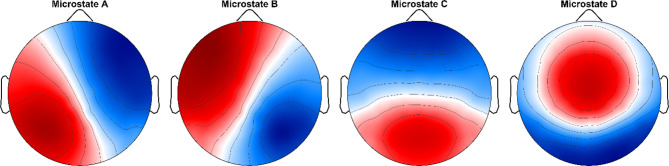
EEG microstate topographies during mind-wandering. These four maps were extracted across MCI patients and healthy older and younger participants.

Based on the cluster analysis^57^ and the optimization criteria^46^, we found four microstates that optimally explained the data across MCI patients and healthy older and younger participants. The extracted maps had the prototypical configurations of right frontal to left posterior, left frontal to right posterior, frontal to occipital, and frontocentral, respectively. These four maps correspond very well with the meta-microstate maps described previously^58^ (Fig. 3).

We then analyzed GEV and temporal parameters of duration, time coverage, and occurrence of the four EEG microstate maps. All temporal parameters were within the normative 95% prediction intervals^59^. As predicted, our results showed significant differences between groups for microstate C and, interestingly, also for microstate A across all parameters.

**Fig. 4:**
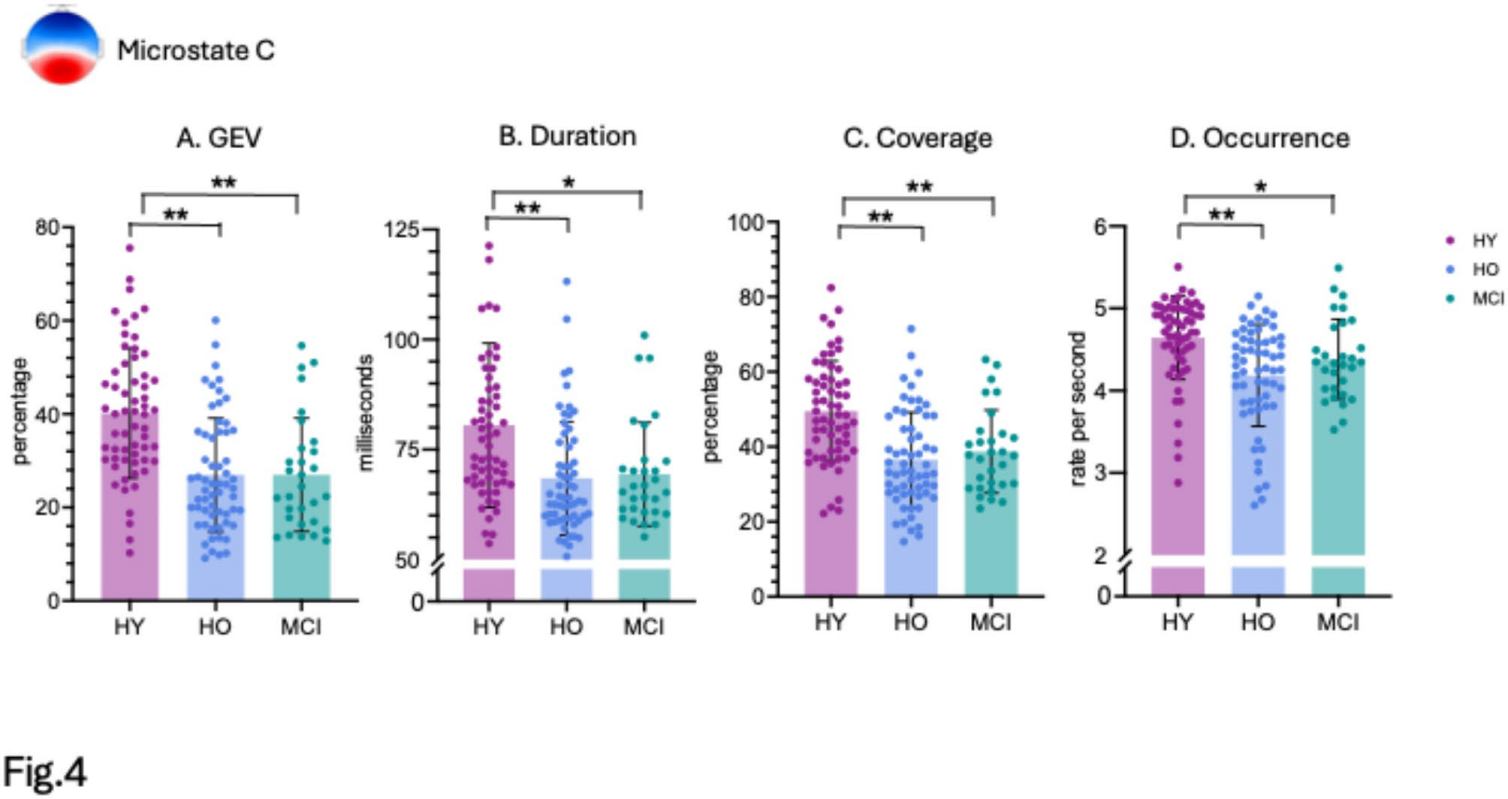
(**A**) EEG microstate C parameters of GEV, (**B**) duration, (**C**) time coverage, and (**D**) occurrence. Error bars indicate standard deviations. P values are FDR corrected. *p < 0.05, **p < 0.005.

For microstate C, we observed decreased GEV values for MCI patients (M = 26.99, SD = 12.15, *p* < 0.001, d = -0.977) and healthy older (M = 26.96, SD = 12.18, *p* < 0.001, d = -1.003) compared to younger participants (M = 40.17, SD = 13.94) (Fig. 4A). Next, we found decreased values for the duration in MCI patients (M = 69.39, SD = 11.80, *p* = 0.011, d = -0.660) and healthy older (M = 68.47, SD = 12.87, *p* < 0.001 d = -0.747) compared to younger participants (M = 80.52, SD = 18.67) (Fig. 4B). Similarly, we show decreased time coverage values for MCI patients (M = 38.73, SD = 11.05, *p* < 0.001, d = -0.845) and healthy older participants (M = 36.50, SD = 12.69, *p* < 0.001, d = -0.992) compared to younger participants (M = 49.51, SD = 13.37) (Fig. 4C). Finally, our data revealed decreased occurrence values in MCI patients (M = 4.38, SD = 0.49, *p* = 0.040, d = -0.518) and healthy older participants (M = 4.18, SD = 0.62, *p* < 0.001, d = -0.806) compared to younger participants (M = 4.64, SD = 0.51) (Fig. 4D).

**Fig. 5:**
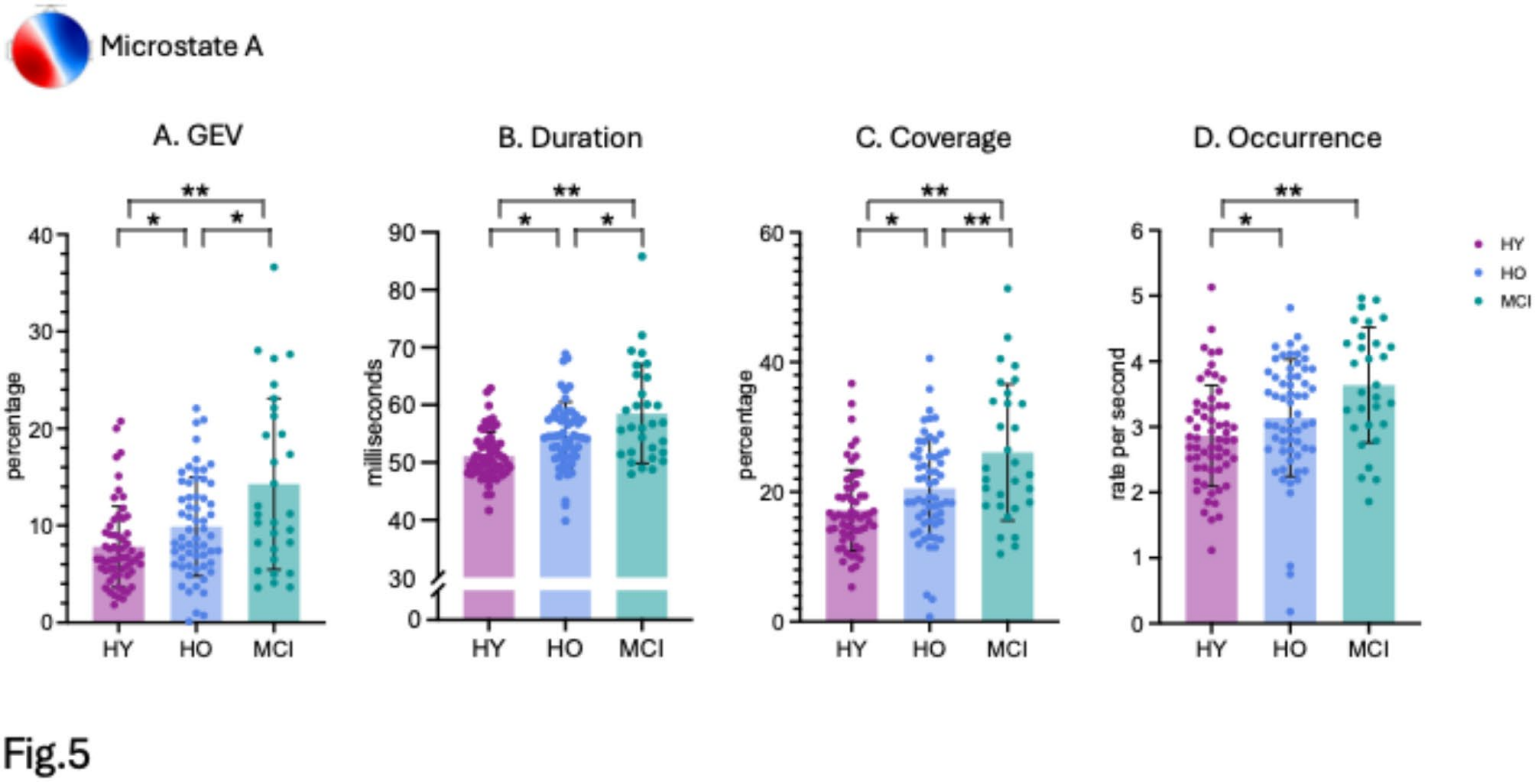
(**A**) EEG microstate A parameters of GEV, (**B**) duration, (**C**) time coverage, and (**D**) occurrence. Error bars indicate standard deviations. P values are FDR corrected. *p < 0.05, **p < 0.005.

For microstate A, we observed increased GEV values for MCI patients (M = 14.29, SD = 8.83) compared to healthy older participants (M = 9.89, SD = 5.10, *p* = 0.011, d = 0.663) and healthy younger (M = 7.82, SD = 4.18, *p* < 0.001, d = 1.049). There was also a significant difference between healthy older and healthy younger participants (*p* = 0.033, d = 0.442) (Fig. 5A). We found increased duration for MCI patients (M = 58.75, SD = 8.61) compared to both healthy older (M = 54.75, SD = 5.74, *p* = 0.033, d = 0.543) and healthy younger participants (M = 51.11, SD = 4.19, *p* < 0.001, d = 1.215). The mean duration was higher for healthy older subjects compared to healthy younger (*p* < 0.001, d = 0.720) (Fig. 5B). Similarly, we observed increased time coverage for MCI patients (M = 26.09, SD = 10.50) compared to healthy older (M = 20.54, SD = 7.70, *p* = 0.015, d = 0.631) and healthy younger participants (M = 17.18, SD = 6.22, *p* < 0.001, d = 1.119). Microstate A time coverage was also higher for healthy older participants compared to healthy younger (*p* = 0.025, d = 0.477) (Fig. 5C). Finally, for occurrence rate, we found increased value for MCI patients (M = 3.64, SD = 0.88) compared to healthy older (M = 3.14, SD = 0.89, *p* = 0.031, d = 0.558) and healthy younger participants (M = 2.87, SD = 0.77, *p* < 0.001, d = 0.947). There was no significant difference between healthy older and younger participants (*p* = 0.118) (Fig. 5D). Detailed results of all EEG microstate parameters for each group can be found in Supplementary material (SI Appendix, Table S3).

### Brain activity in the “Self-experience” memory network decreases in healthy aging population and MCI patients

High-density EEG offers a powerful tool for investigating neural brain dynamics with high temporal and spatial resolution^60^. We have previously demonstrated that even subcortical activity is detectable with hdEEG source imaging^61,62^. Here, we applied a similar EEG source imaging technique on the EEG microstates. Based on our previous findings^46^, we expected that microstate C, which is less present in MCI patients and healthy aging population, is generated by activity in the memory network, particularly involving the hippocampus. Furthermore, in order to associate the psychological, mind-wandering content with the neurophysiological EEG microstates, we correlated the temporal parameters of microstate C with the self-related categories of the ARSQ. We found a significant positive correlation between occurrence of microstate C and the visual thoughts of the behavioral resting state questionnaire (*R* = 0.21, *p* = 0.01), suggesting that the less microstate C occurred, the less the MCI patients were able to picture places and events while mind-wandering. We then localized the sources of each time point labeled with microstate C of each participant, averaged them across all time points, and then across all 150 participants.

**Fig. 6:**
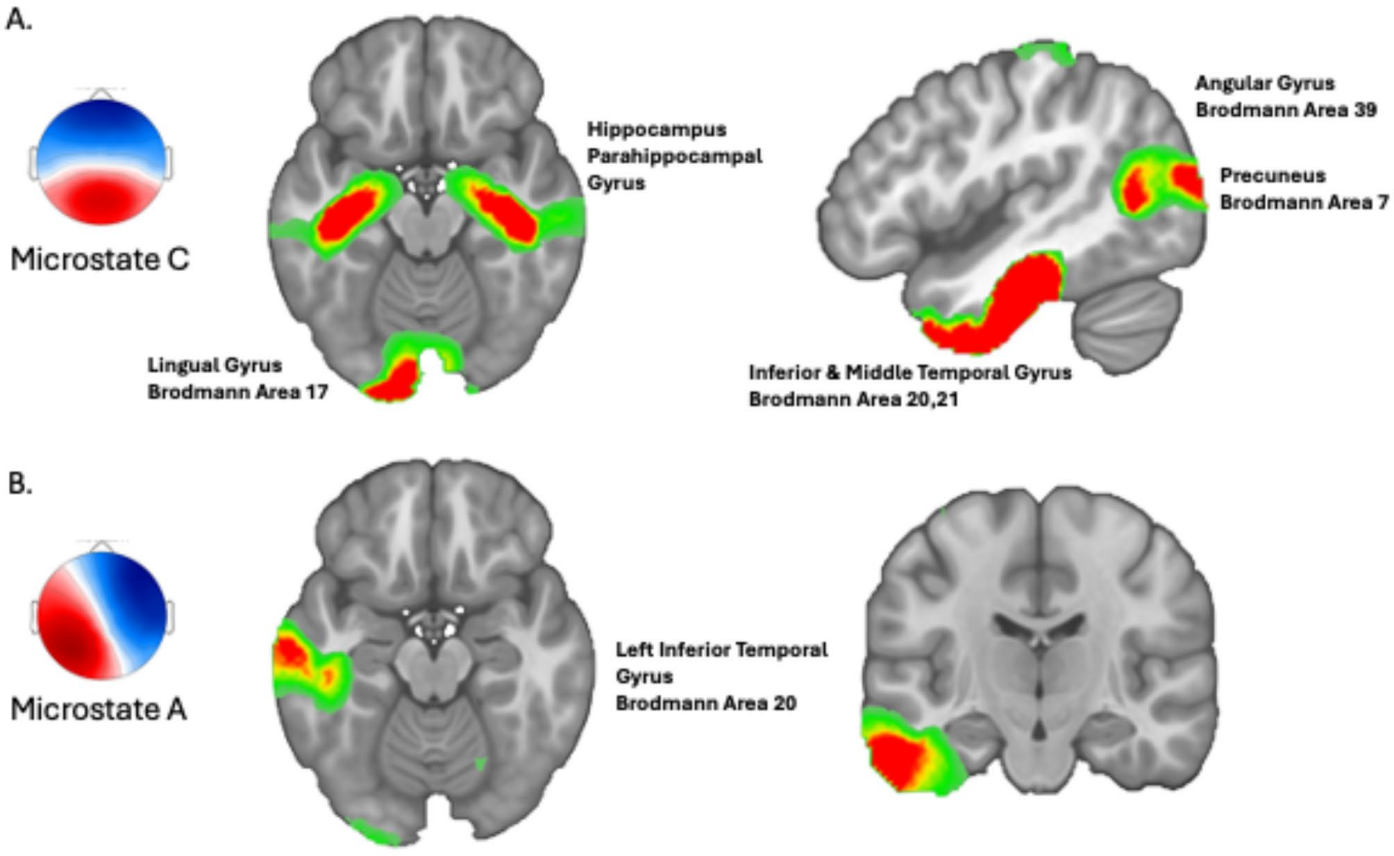
(**A**) Sources of EEG microstate C during mind-wandering. These sources were extracted across MCI patients and healthy older and younger participants. (**B**) Sources of EEG microstate A during mind-wandering. These sources were extracted across MCI patients and healthy older and younger participants.

We found the sources underlying microstate C localized in the hippocampus, the parahippocampal gyrus, the inferior temporal lobe, the primary visual cortex, the precuneus, and the left angular gyrus (Fig. 6A). These areas are known to belong to the default mode network and play a role in autobiographical episodic memory^63^. The main activity for microstate A was found in the left inferior and middle temporal gyrus (Fig. 6B). The supplementary material provides detailed results of source localization of all microstates (SI Appendix, Figure S1).

## Discussion

Here, we provide insights into self-related thoughts and their associated neural substrates in individuals with mild cognitive impairment (MCI). The human ability to re-experience one’s past by subjectively locating oneself in a previously experienced place and time or imagining oneself in the future plays a fundamental role in everyday life. Our findings indicate alterations in self-related thoughts associated with brain electrical activity dysfunction in the key regions of episodic autobiographical memory and default mode networks.

Our neuropsychological findings reveal a significant decline in global cognitive functioning, as measured by the Montreal Cognitive Assessment (MoCA), among MCI patients compared to both healthy older and younger controls. The MoCA, a validated cognitive screening tool designed to identify the MCI population, evaluates multiple cognitive domains, including memory, language, attention, or orientation. Our results confirm the sensitivity of MoCA in detecting MCI patients and effectively distinguish them from healthy controls. Our findings highlight that mild cognitive impairment is distinct from normal cognitive aging. Mild cognitive impairment represents an early stage of memory loss in individuals who can still independently perform most daily activities; however, cognitive changes can be noticed using neuropsychological screening. This pattern was further supported by a significantly lower MoCA memory subscore and the MoCA Memory Index Score (MIS-MoCA) in MCI patients compared to the healthy controls. Notably, the healthy older individuals also differed significantly from the younger participants in the memory MoCA subscore, indicating subtle cognitive deficits. Subsequently, we tested whether these cognitive changes, identified using the standardized MoCA screening tool, were also associated with visible changes in self-related thoughts during mind-wandering.

Similarly, our analysis of the Amsterdam Resting-State Questionnaire (ARSQ) revealed significant alterations in self-related thoughts among MCI patients compared to healthy controls. Specifically, we found a significant reduction in self-related conscious thoughts in MCI patients, a phenomenon not previously measured with the retrospective, subjective questionnaire. The “Self” category captures self-related thoughts, including one’s own feelings and behavior. The “Planning” category refers to future-directed thoughts, such as planning tasks and contemplating the future. The “Visual Thoughts” category corresponds to visual imagery during mind-wandering, including conscious thoughts about specific places and events. Our data indicate a significant decline in self-related thoughts during mind-wandering among both MCI patients and healthy older participants. Interestingly, healthy older individuals also already showed reduced self-related thoughts, though less pronounced than MCI patients, corresponding to the observed decrease in MoCA scores in this population. Subsequently, we tested whether the cognitive changes we observed using the behavioral MoCA and ARSQ screening tools were also evident on the level of intrinsic brain activity.

Previous research has demonstrated that cognitive processes supporting mind-wandering, particularly those involved in episodic memory retrieval and episodic future thinking, are reduced in dementia. Alzheimer’s disease is a progressive neurodegenerative condition characterized by the presence of neurofibrillary tangles and amyloid plaques^64^. Neuroimaging work has determined that this neurodegenerative disease includes brain regions in the prefrontal, temporal, and parietal cortices. These brain regions comprise a neural network crucial for functions of the default mode, episodic memory, and episodic future thinking^65,66^. Given this context, it is not surprising that individuals with Alzheimer’s disease experience disturbances in episodic autobiographical memory for past experiences^7,67,68^ and face challenges in imagining novel scenarios that may occur in the future^13,66,69^.

Thus, the primary objective of our study was to investigate mind-wandering in MCI patients with the anticipation that our results would provide valuable insights into the dysfunction of the cognitive and neural substrates within this clinical population. Previous research by^66^ has shown that amnesic MCI patients exhibit a notable decline in memory for internal episodic details, similar to AD patients, while still being able to employ strategies used by healthy older adults^70^. demonstrated that MCI patients exhibit AD pathology in the medial temporal lobes and anterior cingulate cortex, with the temporal regions largely unaffected. More recently^48^, investigated mind-wandering in both frontotemporal dementia and AD, revealing a connection between altered mind-wandering and structural and functional integrity with the default mode and frontoparietal networks.

In our study, we conducted EEG microstate analysis comparing MCI patients to healthy older and younger individuals to explore potential differences in intrinsic brain network activity during mind-wandering. We identified four data-driven EEG microstates highly resembling topographies reported in the previous literature^71^. Our data indicated a significant decline in GEV and temporal parameters of EEG microstate C during mind-wandering among both MCI patients and healthy older participants compared to healthy young participants. Interestingly, we found a significant increase in GEV and temporal parameters of EEG microstate A during mind-wandering, specifically in MCI patients only. Increase in microstate A in MCI and/or AD patients has previously been reported^23,72–75^ while decrease of microstate C in MCI or AD has been found in^35,39,76^. The observed decrease of microstate C in both MCI patients and healthy older participants raises an interesting question. It may be that the healthy older controls may not all represent a normal aging population, but that some of them have (though undiagnosed) mild cognitive impairment. Further investigations into the aging population would be important in order to disentangle the healthy from pathological aging.

Subsequently, we examined the underlying sources of these four EEG microstates. We hypothesized that the neural network underlying microstate C would reflect the brain regions associated with self-relevant episodic memory processes and overlap with the dysfunctional default mode network. Our previous work has explored the spatiotemporal dynamics of large-scale networks in healthy young participants, confirming the association of the default mode network with conscious experiences during episodic autobiographical memories^46^. Furthermore, recent research^41^ has suggested that microstate C is related to self-referential, personally significant information processing and autobiographical memory. Source analysis of microstate C identified both hippocampi as strong contributors to the scalp topography of microstate C with additional activity in visual areas and the left angular gyrus.

In addition, here we also reported decreased values on Self, Planning, and Visual thought categories of the ARSQ, which reflect the diminished “present self,” “future self,” and “self-imagery” of MCI patients^77^ conducted a study investigating aspects of autobiographical memory, mental imagery, and self-location to assess personal past and future self-location. The authors observed increased activity in a network encompassing temporal, parietal, and occipital cortices when healthy young participants remembered themselves in the past or imagined themselves in the future. Furthermore, we directly tested the relationship between self-categories of the ARSQ and EEG neurophysiology and found positive correlation between “self-imagery” and occurrence of microstate C in MCI patients. These findings align with our hypothesis that autonoetic consciousness, the mental ability to subjectively travel in time, is essential for healthy episodic autobiographical memory and mind-wandering.

High-density EEG offers a powerful approach to investigating neural brain dynamics with exceptional temporal resolution. By using source analysis methods to scalp recordings, it becomes feasible to reconstruct neuronal activities in specific brain regions with millisecond precision, allowing for real-time imaging of the temporal dynamics of whole-brain neuronal networks^60^. Although the accuracy and precision of localizing neuronal activity using EEG techniques remain subjects of ongoing debate, we have recently shown the detectability of subcortical activity with hdEEG source imaging^61^.

Our study reveals contrasting patterns in the microstates during spontaneous mentation between MCI patients and healthy older and younger controls. While there is a notable decrease in microstate C activity in MCI patients and healthy older participants compared to younger participants, we observed a significant increase in the temporal parameters of microstate A among MCI patients compared to healthy controls. This finding aligns with previous research documenting elevated microstate A activity in MCI patients and Alzheimer’s disease patients^72,73^. Source localization analysis identified significant activity in the left inferior and middle temporal gyrus, consistent with prior studies employing EEG source imaging or EEG-fMRI techniques^45,46,78^. Our review paper on the functional significance of microstates highlights the association of microstate A with auditory and verbal functions^79^. Therefore, we posit that the decrease in microstate C, indicative of self-related thoughts, in MCI patients may be compensated for by heightened verbal thoughts. This suggests a potential adaptive mechanism in response to cognitive decline. It is important to note that we have excluded the possibility of our results being due to decreased mood in MCI patients using the Hospital Anxiety and Depression Scale (HADS-A, HADS-B).

In our present hdEEG study, we applied a similar EEG source imaging technique as in^61^ and showed decreased activity, particularly in the hippocampus and parahippocampal gyrus, during spontaneous mentation of MCI patients. Our findings reveal the brain networks underlying microstate C and underscore the active role of key regions of episodic autobiographical memory and default mode networks in self-relevant, internal thoughts of healthy individuals compared to individuals with memory decline. In our fMRI meta-analysis^4^, we suggested that the lateral parietal cortex and adjacent parts of the temporal cortex are crucial for both the conscious experience of past life episodes (episodic autobiographical memory) and associated with first-person bodily views (bodily self-consciousness). Interestingly, we did not find here activations in the frontal regions, similar to^48^, who recently showed that mind-wandering was associated with stronger left PCC-left posterior hippocampal connectivity compared to frontal activations. Taken together, our findings suggest that the posterior part of memory and default mode networks may be particularly relevant for the conscious experience of self in time during mind-wandering. These findings establish a link between spontaneous brain activity in the hippocampus and processes such as episodic autobiographical memory recall and episodic future thinking, which engage the activity in the default mode network. Notably, recent evidence supporting the role of the hippocampus in mind-wandering has been provided by studies involving patients with bilateral hippocampal damage^80^.

## Limitations

Our study presents several limitations that should be addressed in future work. First, we did not perform complex neuropsychological examinations of the healthy older and younger participants. Our neurophysiological results show that deeper investigations into the aging population would be relevant to gain better understanding of the underlying differences between the healthy and pathological aging. Another limitation of this current work is the use of the MNI head model for source localization. It is preferrable to use the individual MRI of the participant to construct the individual head model^81^. A template MRI can be used (here, we used the MNI brain), but the source localization is less precise. An additional limitation in our work is the absence of correlations between Self and Planning categories of the ARSQ with the EEG microstate C of MCI patients. While we found a correlation between microstate C and Visual thoughts of ARSQ, it would have been even stronger evidence to see a correlation with the other self categories. Further, the ARSQ was translated by bilingual researchers, although, at this stage the French version of ARSQ has not been officially validated.

## Conclusions

Our study employs fast temporal resolution electrical brain imaging to assess mind-wandering in individuals with mild cognitive impairment (MCI). Our findings reveal altered patterns of self-related thoughts in MCI patients compared to healthy controls. These changes in mind-wandering patterns were associated with decreased activity in the hippocampus, angular gyrus, inferior temporal lobe, precuneus, and visual cortices. Given the ubiquitous nature of mind-wandering in daily life, it becomes crucial to understand how the loss of this fundamental human capacity impacts the sense of self in dementia patients. Understanding the implications of these alterations in mind-wandering patterns can offer valuable insights into the lived experiences of individuals coping with cognitive decline and may guide the development of interventions aimed at preserving cognitive function and enhancing the quality of life in dementia patients^82^.

## Materials and methods

### Participants

We enrolled 150 participants in this study: 30 amnestic MCI patients (13 females; mean age: M = 71.13 years, SD = 7.92 years), 60 healthy old-aged participants (34 females; mean age: M = 71.27 years, SD = 7.92 years), and 60 healthy younger participants (33 females; mean age: M = 25.93 years, SD = 7.92 years). After each EEG recording session, 108 participants: 29 MCI patients (13 female; mean age: M = 71 years, SD = 6.5 years), 49 healthy older participants (29 female; mean age: M = 72.4 years, SD = 7.3 years) and 30 healthy young participants (16 female; mean age: M = 25.3 years, SD = 3.1 years) completed the Amsterdam Resting-State questionnaire. The study was approved by the Cantonal Ethical Committee of Geneva (CCER). All participants gave their written informed consent to participate in accordance with the CCER and the Declaration of Helsinki (2013).

### MCI patients’ recruitment, inclusion and exclusion criteria

The MCI patients’ recruitment took place at the Memory Center of the Geneva University Hospitals (HUG) in Geneva. Diagnosis of MCI was performed by specialized neurologists. A trained clinical psychologist contacted by phone and sent information letter to those MCI patients who showed an interest in participating in the study. Further, the clinical psychologist performed detailed check concerning the inclusion criteria: age 55+; confirmation of clinical diagnosis of MCI done by the study MD; Mini-Mental State Exam ≥ 18; minimum of completed 8th-grade education; on a stable dose of medications for memory loss including cholinesterase inhibitors (e.g. donepezil, rivastigmine) or memantine as defined as 6 consecutive weeks of treatment at an unchanging dose; willing and capable to give informed consent for participation in the study after it has been thoroughly explained) and exclusion criteria: any current diagnosis of a major psychiatric disorder (e.g., schizophrenia, bipolar disorder, major depressive disorder); any history of other progressive or genetic neurologic disorder (e.g. Parkinson’s disease, multiple sclerosis, tubular sclerosis) or acquired neurological disease (e.g. stroke, traumatic brain injury, tumor), including intracranial lesions; history of head trauma resulting in prolonged loss of consciousness; history of poorly controlled headaches; history of seizures, diagnosis of epilepsy; any unstable medical condition; contraindications to tACS and MRI.

## MCI patients: neuropsychological examination

An extensive neuropsychological test battery assessing global cognition (Mini-Mental State Examination, Clock Drawing test, Three objects-three places, Montreal Cognitive Assessment), episodic memory (Selective Reminding Test-immediate, free and cued, Rey–Osterrieth Complex Figure- copy and recall, Logical Memory Story B- immediate and delayed, Memory Index Score), working memory (Digit Span), language (semantic fluency (fruits), phonemic fluency (V)), attention (Coding), executive functions (Trail Making Tests A-B). Further, we also tested the psychological health, particularly depression and anxiety using the Hospital Anxiety and Depression Scale (HADS-A and HADS-D). Detailed results of all neuropsychological test for MCI patients can be found in Supplementary material (SI Appendix, Table S4).

## Neuropsychological testing

### Montreal cognitive assessment (MoCA)

The Montreal Cognitive Assessment (MoCA) is a well-validated, highly sensitive screening tool for the early detection of MCI^24,83^. The cognitive battery examines various tasks across multiple domains, including visuospatial, executive functioning, attention, language, memory, and orientation. A composite score is derived by summing up scores from these domains, with the maximum achievable MoCA score being 30 points. Lower scores on the MoCA indicate a more significant cognitive decline, and 26 or below typically signify cognitive impairment. All MCI patients were French speaking, therefore we used the French version of MoCA and used three different versions 8.1, 8.2 and 8.3.

### Memory index score (MIS)

The Memory Index Score (MIS) measures delayed memory recall, a component that can be derived from the Montreal Cognitive Assessment (MoCA). Unlike the MoCA, which solely evaluated the delayed free recall, the MIS evaluates both free and cued recall. The score ranges from 0 to 15^84^. Specifically, participants are asked to recall five unrelated words after a 5-minute delay. Three points per word are given for each word freely recalled without a cue, 2 points per word are given for each word recalled with a categorical clue, and 1 point per word is given for each word recalled with a multiple-choice option. While only minimal changes may be seen in the overall MoCA scores, the MIS can reveal significant effects, particularly in assessing delayed memory recall abilities.

### Amsterdam resting-state questionnaire (ARSQ)

To measure the thoughts and feelings experienced by participants during rest, we administered the Amsterdam Resting-State Questionnaire (ARSQ, version 2.0 – translated into French)^49^. Participants rated 30 statements about their emotions and thoughts during the EEG resting-state session on a Likert-type scale ranging from 1 (completely disagree) to 5 (completely agree). The ARSQ covers ten domains of resting-state cognition: Discontinuity of Mind, Theory of Mind, Self, Planning, Sleepiness, Comfort, Somatic Awareness, Health Concern, Visual Thought and Verbal Thought. Each domain is evaluated with three statements. We were particularly interested in three domains: (i) Self (“I thought about myself,” “I thought about my behavior,” and “I thought about my feelings”), (ii) Planning (“I thought about the future,” “I thought about things I need to do,” “I thought about solving problems”), and (iii) Visual Thought (“I pictured places,” “I pictured events,” “I thought in images”). We calculated the scores of each ARSQ domain by taking the sum of three statements.

### EEG data recording and pre-processing

During the experiment, participants were comfortably seated in a dimly lighted, attenuated, and electrically shielded room and instructed to keep their eyes closed and to relax. Five minutes of eyes-closed resting-state EEG data were collected using 257 channels EEG channels (HydroCel Geodesic Sensor Net; Magstim EGI Eugene, USA), and all electrodes were referenced against vertex. Data was sampled at 1000 Hz between DC and 100 Hz. A trained research assistant monitored sleepiness online during the EEG recordings in order to control vigilance fluctuations and ensure that the participants did not fall asleep. Furthermore, we have rated the “Comfort” and “Sleepiness” domains of each participants using the ARSQ. There were no differences between groups on those subscores.

The offline EEG data processing was conducted using the Cartool toolbox^57^ and MATLAB. Channels located on the neck and cheeks were excluded from the analysis. The data were downsampled to 250 Hz and bandpass filtered between 1 and 40 Hz using Butterworth filter of 2nd order with a − 12 dB/octave roll-off. The filtering was computed linearly forward and backward to eliminate the phase shift. Artifacts caused by eye movements and heartbeat were corrected using infomax-based ICA^85^ using an in-house Matlab script. Channels with excessive artifacts were manually rejected and then interpolated using the 3D spherical spline method^86^. Finally, the reference was re-projected to average.

### Microstates segmentation

Microstate analysis was conducted in two stages: individual and group levels. First, topographies at global field power peaks were submitted to k-means clustering for cluster solutions ranging from 1 to 12 while ignoring polarity. Spatial filtering was applied to spatially smooth individual EEGs^81^. A minimum correlation threshold (≤ 0.5) was used, and topographies failing to reach this threshold remained unassigned. To maximize global explained variance (GEV), clusters were iterated 100 times. The optimal set of clusters for each participant was based on the meta-criteria implemented in Cartool^45,46^.

In the second analysis stage, separate k-means analyses were performed for each group to spatially compare group-level topographies of MCI patients and healthy older and younger participants. Polarity differences were ignored, and a minimal spatial correlation threshold of 0.5 was applied. The clusters ranged from 1 to 15, with 200 iterations for each cluster solution. The optimal number of group-level clusters was based on the meta-criteria. Since separate subgroup maps result in substantially inflated false positive error rates^87^, the second k-means clustering was conducted by submitting the most dominant topographies of all participants while maintaining the same parameters. The meta-criterium revealed four maps that optimally explained all the data. These optimal four microstate maps across all participants were used for backfitting. Detailed results of all EEG microstates for each group can be found in Supplementary material (SI Appendix, Figure S2).

### Backfitting

The four group-level topographical maps obtained from all participants were backfitted to the original individual EEGs using the winner-takes-all approach. This involved calculating the spatial correlation between the group-level maps and topography of each time frame (not only GFP peaks) of individual EEG. Polarity was ignored, and a minimal correlation threshold of 0.5 was applied. Temporal smoothing was implemented using a sliding window size of 11 (5 time frames (20 msec) on each side of the current time frame) and a weight smoothness factor of 15. Segments smaller than or equal to 5-time frames (20 ms) were rejected and split in half, with the first half added to the preceding segment and the second added to the subsequent segment.

Duration (calculated in ms; mean length of the segments), occurrence rate (number of segments divided by the total labeled duration), and time coverage (calculated in %; percentage of time covered by one microstate) were calculated for each microstate class. GEV (calculated in %; the sum of the explained variances weighted by the Global Field Power (GFP) at each moment in time) for each microstate was calculated by summing the squared spatial correlations between the group-level topographical map and its corresponding assigned maps at each time point, weighted by the GFP^88^. The GEV of each microstate was then summed, providing the data portion explained by group-level microstates.

### EEG source localization

We calculated a distributed linear inverse solution (LORETA)^81,89^ to estimate the sources contributing to each microstate map. The lead field for the inverse solution was calculated for 204 electrode positions and the average brain of the Montreal Neurological Institute (MNI) in a grey matter-constrained head model using the LSMAC head model with 6926 equally distributed solution points (6 mm distance between points)^81^. Each solution point was standardized across time to eliminate activation biases. The estimated current densities of each participant corresponding to periods labelled by a given EEG microstate were averaged across all time points. Only time points with correlations higher than 0.9 between data topography and microstate topography were considered.

### Statistical analysis

One-way ANOVA was used to compare EEG microstate parameters between the three groups (MCI patients, healthy older and healthy younger participants), followed by independent samples t-test post-hoc tests for pairwise comparisons. All post-hoc comparisons were adjusted for multiple testing using False Discovery Rate (FDR, α = 0.05)^90^. Effect sizes for pairwise comparisons were reported using Cohen’s d.

## Electronic supplementary material

Below is the link to the electronic supplementary material.

Supplementary Material 1

Supplementary Material 2

## Data Availability

The datasets analyzed during the current study are available from the corresponding author upon reasonable request.
