# Supplementary material for: Self-related thought alterations associated with intrinsic brain dysfunction in mild cognitive impairment
Source: Sci Rep. 2025 Apr 10;15:12279. doi: 10.1038/s41598-025-97240-8 (PMC11986127; doi:10.1038/s41598-025-97240-8)

**Supplementary material and results**

**S1. MoCA subscores**

Table S1. Mean values and standard deviations for each group and each MoCA subscore and statistical analysis results. P values for pairwise comparisons are FDR corrected.

|  | Mean and ± standard deviation | | | ANOVA df(2,147) | | | MCI vs Older | | MCI vs Younger | | Older vs Younger | |
| --- | --- | --- | --- | --- | --- | --- | --- | --- | --- | --- | --- | --- |
|  | **MCI**  **(n=30)** | **Older (n=60)** | **Younger (n=60)** | **F value** | **p value** | | **p value** | **Cohen’s d** | **p value** | **Cohen’s d** | **p value** | **Cohen’s d** |
| Visuospatial | 3.27±0.87 | 3.25±0.77 | 3.53±0.70 | 2.377 | | 0.096 | - | - | - | - | - | - |
| Executive | 3.17±0.75 | 3.57±0.70 | 3.87±0.43 | 13.11 | | <0.001 | 0.028 | -0.555 | <0.001 | -1.125 | 0.012 | -0.514 |
| Attention | 5.37±1.13 | 5.55±0.68 | 5.75±0.60 | 1.56 | | 0.070 | - | - | - | - | - | - |
| Language | 4.47±0.78 | 4.60±0.64 | 4.80±0.51 | 3.21 | | 0.043 | 0.520 | -0.192 | 0.032 | -0.539 | 0.107 | -0.341 |
| Orientation | 5.30±0.06 | 5.92±0.28 | 5.87±0.34 | 14.22 | | <0.001 | <0.001 | -0.944 | <0.001 | -0.841 | 0.520 | 0.159 |
| Memory | 1.87±1.96 | 3.85±1.34 | 4.15±1.19 | 27.35 | | <0.001 | <0.001 | -1.252 | <0.001 | -1.520 | 0.315 | -0.235 |
| MIS | 9.00±4.19 | 12.67±3.06 | 13.28±2.74 | 19.07 | | <0.001 | <0.001 | -1.046 | <0.001 | -1.291 | 0.371 | -0.211 |
| Overall | 23.40±4.15 | 26.73±2.56 | 27.97±1.85 | 28.41 | | <0.001 | <0.001 | -1.104 | <0.001 | -1.604 | 0.007 | -0.549 |

**S2. ARSQ scores**

Table S2. Mean values and standard deviations for each group and each ARSQ domain and statistical analysis results. P values for pairwise comparisons are FDR corrected only for domains of Self, Planning, and Visual thought (see the main text).

|  | Mean and ± standard deviation | | | ANOVA df(2,105) | | | MCI vs Older | | MCI vs Younger | | Older vs Younger | |
| --- | --- | --- | --- | --- | --- | --- | --- | --- | --- | --- | --- | --- |
|  | **MCI**  **(n=29)** | **Older (n=49)** | **Younger (n=30)** | **F value** | **p value** | | **p value** | **Cohen’s d** | **p value** | **Cohen’s d** | **p value** | **Cohen’s d** |
| Self | 8.14±2.81 | 8.92±2.81 | 10.50±2.84 | 4.57 | | 0.013 | 0.313 | -0.242 | 0.011 | -0.729 | 0.027 | -0.556 |
| Planning | 6.45±3.34 | 6.76±3.30 | 10.30±2.69 | 14.64 | | <0.001 | 0.165 | -0.341 | <0.001 | -1.254 | <0.001 | -1.137 |
| Visual | 7.62±3.76 | 7.20±3.76 | 11.24±3.25 | 9.38 | | <0.001 | 0.581 | 0.126 | 0.002 | -1.001 | <0.001 | -1.099 |
| Sleepiness | 4.35±1.68 | 4.63±2.48 | 6.50±2.97 | 7.17 | | 0.0012 | 0.731 | -0.076 | <0.001 | -1.054 | <0.001 | -1.131 |
| Comfort | 10.86±2.86 | 11.67±2.85 | 11.47±1.74 | 0.90 | | 0.408 | - | - | - | - | - | - |
| Health | 4.79±2.32 | 4.22±1.55 | 4.63±2.39 | 0.82 | | 0.443 | - | - | - | - | - | - |
| DoM | 7.59±6.69 | 7.00±2.86 | 8.73±2.48 | 1.61 | | 0.204 | - | - | - | - | - | - |
| ToM | 6.66±3.03 | 7.14±3.21 | 9.07±2.68 | 5.50 | | 0.005 | 0.425 | -0.177 | 0.004 | -0.894 | 0.016 | -0.658 |
| Verbal | 7.07±3.05 | 6.57±2.92 | 8.81±3.31 | 4.01 | | 0.021 | 0.503 | 0.153 | 0.007 | -0.821 | <0.001 | -1.011 |
| SA | 8.86±2.86 | 9.49±2.84 | 8.93±2.73 | 0.60 | | 0553 | - | - | - | - | - | - |

Verbal – Verbal thought; Health – Health concern; DoM- Discontinuity of Mind; ToM – Theory of Mind; Verbal – Verbal thought; SA – Somatic awareness

**S3. EEG microstates parameters**

Table S3. Mean values and standard deviations for each group and each microstate parameter and statistical analysis results. P values for pairwise comparisons are FDR corrected.

|  |  | Mean and ± standard deviation | | | ANOVA df(2,147) | | MCI vs Older | | MCI vs Younger | | Older vs Younger | |
| --- | --- | --- | --- | --- | --- | --- | --- | --- | --- | --- | --- | --- |
|  |  | **MCI**  **(n=30)** | **Older (n=60)** | **Younger (n=60)** | **F value** | **p value** | **p value** | **Cohen’s d** | **p value** | **Cohen’s d** | **p value** | **Cohen’s d** |
| GEV % | **MS A** | 14.29±8.83 | 9.89±5.10 | 7.82±4.18 | 12.75 | <0.001 | 0.011 | 0.663 | <0.001 | 1.049 | 0.033 | 0.442 |
|  | **MS B** | 8.77±4.40 | 11.76±5.53 | 10.77±6.62 | 2.66 | 0.073 | - | - | - | - | - | - |
|  | **MS C** | 26.99±12.15 | 26.96±12.18 | 40.17±13.94 | 18.81 | <0.001 | 1 | 0.002 | <0.001 | -0.977 | <0.001 | -1.003 |
|  | **MS D** | 7.99±7.11 | 12.37±10.59 | 7.21±9.18 | 4.88 | 0.009 | 0.087 | -0.453 | 0.921 | 0.089 | 0.015 | 0.518 |
| Duration | **MS A** | 58.11±4.19 | 54.75±5.75 | 51.11±4.19 | 16.31 | <0.001 | 0.033 | 0.543 | <0.001 | 1.215 | <0.001 | 0.720 |
|  | **MS B** | 53.26±5.45 | 56.21±5.16 | 53.48±7.16 | 3.83 | 0.024 | 0.032 | -0.556 | 1 | -0.032 | 0.036 | 0.436 |
|  | **MS C** | 69.39±11.80 | 68.47±12.87 | 80.52±18.67 | 10.65 | <0.001 | 0.965 | 0.073 | 0.011 | -0.660 | <0.001 | -0.747 |
|  | **MS D** | 51.56±8.98 | 55.71±12.58 | 47.24±10.84 | 8.43 | <0.001 | 0.186 | -0.357 | 0.118 | 0.417 | <0.001 | 0.717 |
| Cover-age | **MS A** | 26.09±10.50 | 20.54±7.70 | 17.18±6.22 | 13.04 | <0.001 | 0.015 | 0.631 | <0.001 | 1.119 | 0.025 | 0.477 |
|  | **MS B** | 18.83±6.94 | 22.42±7.50 | 20.39±8.62 | 2.29 | 0.104 | - | - | - | - | - | - |
|  | **MS C** | 38.73±11.05 | 36.50±12.69 | 49.51±13.37 | 17.29 | <0.001 | 0.588 | 0.181 | <0.001 | -0.845 | <0.001 | -0.992 |
|  | **MS D** | 16.92±12.49 | 20.88±14.72 | 12.92±13.81 | 4.84 | 0.009 | 0.316 | -0.279 | 0.286 | 0.296 | 0.011 | 0.554 |
| Occurrence | **MS A** | 3.64 ± 0.88 | 3.14 ± 0.89 | 2.86 ± 0.77 | 8.40 | <0.001 | 0.031 | 0.558 | <0.001 | 0.947 | 0.130 | 0.325 |
|  | **MS B** | 3.01 ± 0.80 | 3.35 ± 0.79 | 3.17 ± 0.81 | 1.97 | 0.143 | - | - | - | - | - | - |
|  | **MS C** | 4.38 ± 0.49 | 4.18 ± 0.62 | 4.64 ± 0.51 | 10.45 | <0.001 | 0.203 | 0.338 | 0.040 | -0.518 | <0.001 | 0.806 |
|  | **MS D** | 2.57 ± 1.52 | 2.81 ± 1.44 | 1.94 ± 1.70 | 4.74 | 0.010 | 0.641 | -0.164 | 0.161 | 0.376 | 0.011 | 0.546 |

**S4. EEG source localization**

Figure S1. **Estimated sources of the four microstates during mind-wandering.** A distributed linear inverse solution (Loreta) using the MNI template brain was applied to each time point that was labelled with a given microstate, thresholded at >90% correlation. The sources were normalized by the mean activity over the whole EEG for each solution point. The source maps were then averaged across all time points for each microstate. The figure shows the average across the 150 participants.

**
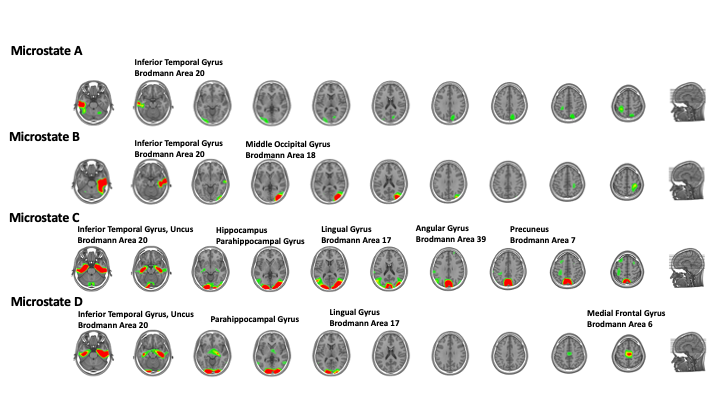
**

**S5. EEG microstates**

Figure S2. Group-level maps were estimated by separately clustering MCI patients, healthy older and healthy younger participants, and all subjects combined (All).


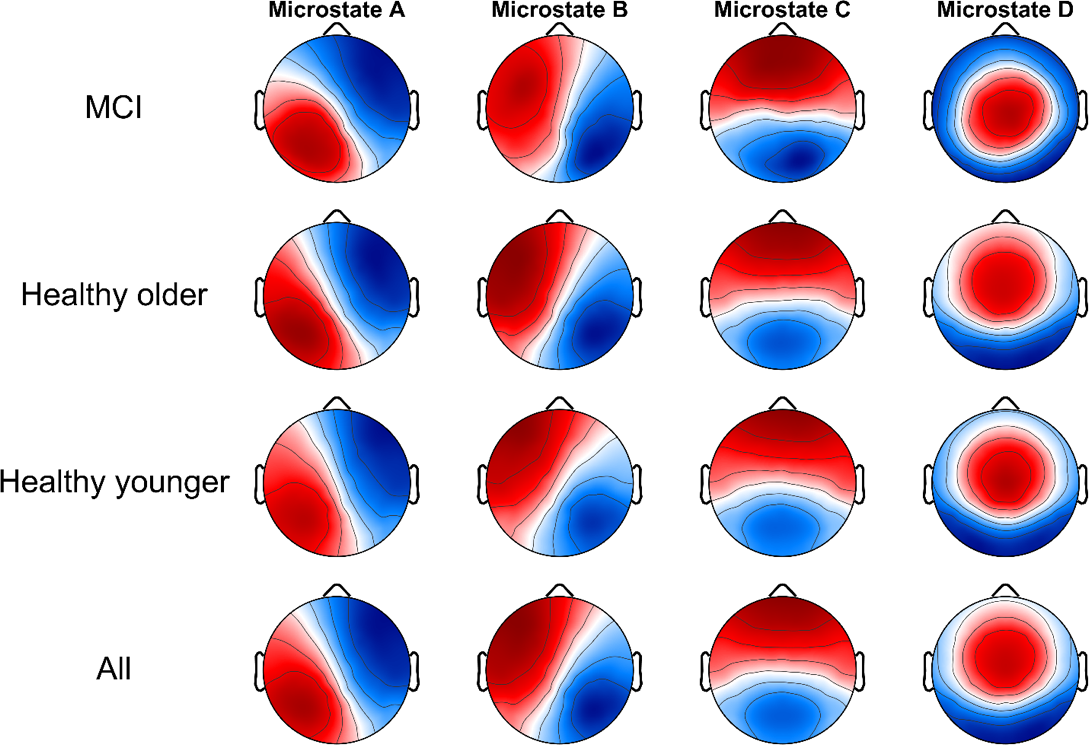

Supplement: Supplementary file 1 — Supplementary Material 1 [file 41598_2025_97240_MOESM1_ESM.docx]
