# Supplementary material for: Self-related thought alterations associated with intrinsic brain dysfunction in mild cognitive impairment
Source: Sci Rep. 2025 Apr 10;15:12279. doi: 10.1038/s41598-025-97240-8 (PMC11986127; doi:10.1038/s41598-025-97240-8)
Supplement: Supplementary file 2 — Supplementary Material 2 [file 41598_2025_97240_MOESM2_ESM.docx]

**Supplementary material and results**

**S1. MoCA subscores**

Table S1. Mean values and standard deviations for each group and each MoCA subscore and statistical analysis results. P values for pairwise comparisons are FDR corrected.

|  | Mean and ± standard deviation | | | ANOVA df(2,147) | | | MCI vs Older | | MCI vs Younger | | Older vs Younger | |
| --- | --- | --- | --- | --- | --- | --- | --- | --- | --- | --- | --- | --- |
|  | **MCI**  **(n=30)** | **Older (n=60)** | **Younger (n=60)** | **F value** | **p value** | | **p value** | **Cohen’s d** | **p value** | **Cohen’s d** | **p value** | **Cohen’s d** |
| Visuospatial | 3.27±0.87 | 3.25±0.77 | 3.53±0.70 | 2.377 | | 0.096 | - | - | - | - | - | - |
| Executive | 3.17±0.75 | 3.57±0.70 | 3.87±0.43 | 13.11 | | <0.001 | 0.028 | -0.555 | <0.001 | -1.125 | 0.012 | -0.514 |
| Attention | 5.37±1.13 | 5.55±0.68 | 5.75±0.60 | 1.56 | | 0.070 | - | - | - | - | - | - |
| Language | 4.47±0.78 | 4.60±0.64 | 4.80±0.51 | 3.21 | | 0.043 | 0.520 | -0.192 | 0.032 | -0.539 | 0.107 | -0.341 |
| Orientation | 5.30±0.06 | 5.92±0.28 | 5.87±0.34 | 14.22 | | <0.001 | <0.001 | -0.944 | <0.001 | -0.841 | 0.520 | 0.159 |
| Memory | 1.87±1.96 | 3.85±1.34 | 4.15±1.19 | 27.35 | | <0.001 | <0.001 | -1.252 | <0.001 | -1.520 | 0.315 | -0.235 |
| MIS | 9.00±4.19 | 12.67±3.06 | 13.28±2.74 | 19.07 | | <0.001 | <0.001 | -1.046 | <0.001 | -1.291 | 0.371 | -0.211 |
| Overall | 23.40±4.15 | 26.73±2.56 | 27.97±1.85 | 28.41 | | <0.001 | <0.001 | -1.104 | <0.001 | -1.604 | 0.007 | -0.549 |

**S2. ARSQ scores**

Table S2. Mean values and standard deviations for each group and each ARSQ domain and statistical analysis results. P values for pairwise comparisons are FDR corrected only for domains of Self, Planning, and Visual thought (see the main text).

|  | Mean and ± standard deviation | | | ANOVA df(2,105) | | | MCI vs Older | | MCI vs Younger | | Older vs Younger | |
| --- | --- | --- | --- | --- | --- | --- | --- | --- | --- | --- | --- | --- |
|  | **MCI**  **(n=29)** | **Older (n=49)** | **Younger (n=30)** | **F value** | **p value** | | **p value** | **Cohen’s d** | **p value** | **Cohen’s d** | **p value** | **Cohen’s d** |
| Self | 8.14±2.81 | 8.92±2.81 | 10.50±2.84 | 4.57 | | 0.013 | 0.313 | -0.242 | 0.011 | -0.729 | 0.027 | -0.556 |
| Planning | 6.45±3.34 | 6.76±3.30 | 10.30±2.69 | 14.64 | | <0.001 | 0.165 | -0.341 | <0.001 | -1.254 | <0.001 | -1.137 |
| Visual | 7.62±3.76 | 7.20±3.76 | 11.24±3.25 | 9.38 | | <0.001 | 0.581 | 0.126 | 0.002 | -1.001 | <0.001 | -1.099 |
| Sleepiness | 4.35±1.68 | 4.63±2.48 | 6.50±2.97 | 7.17 | | 0.0012 | 0.731 | -0.076 | <0.001 | -1.054 | <0.001 | -1.131 |
| Comfort | 10.86±2.86 | 11.67±2.85 | 11.47±1.74 | 0.90 | | 0.408 | - | - | - | - | - | - |
| Health | 4.79±2.32 | 4.22±1.55 | 4.63±2.39 | 0.82 | | 0.443 | - | - | - | - | - | - |
| DoM | 7.59±6.69 | 7.00±2.86 | 8.73±2.48 | 1.61 | | 0.204 | - | - | - | - | - | - |
| ToM | 6.66±3.03 | 7.14±3.21 | 9.07±2.68 | 5.50 | | 0.005 | 0.425 | -0.177 | 0.004 | -0.894 | 0.016 | -0.658 |
| Verbal | 7.07±3.05 | 6.57±2.92 | 8.81±3.31 | 4.01 | | 0.021 | 0.503 | 0.153 | 0.007 | -0.821 | <0.001 | -1.011 |
| SA | 8.86±2.86 | 9.49±2.84 | 8.93±2.73 | 0.60 | | 0553 | - | - | - | - | - | - |

Verbal – Verbal thought; Health – Health concern; DoM- Discontinuity of Mind; ToM – Theory of Mind; Verbal – Verbal thought; SA – Somatic awareness

**S3. EEG microstates parameters**

Table S3. Mean values and standard deviations for each group and each microstate parameter and statistical analysis results. P values for pairwise comparisons are FDR corrected.

|  |  | Mean and ± standard deviation | | | ANOVA df(2,147) | | MCI vs Older | | MCI vs Younger | | Older vs Younger | |
| --- | --- | --- | --- | --- | --- | --- | --- | --- | --- | --- | --- | --- |
|  |  | **MCI**  **(n=30)** | **Older (n=60)** | **Younger (n=60)** | **F value** | **p value** | **p value** | **Cohen’s d** | **p value** | **Cohen’s d** | **p value** | **Cohen’s d** |
| GEV % | **MS A** | 14.29±8.83 | 9.89±5.10 | 7.82±4.18 | 12.75 | <0.001 | 0.011 | 0.663 | <0.001 | 1.049 | 0.033 | 0.442 |
|  | **MS B** | 8.77±4.40 | 11.76±5.53 | 10.77±6.62 | 2.66 | 0.073 | - | - | - | - | - | - |
|  | **MS C** | 26.99±12.15 | 26.96±12.18 | 40.17±13.94 | 18.81 | <0.001 | 1 | 0.002 | <0.001 | -0.977 | <0.001 | -1.003 |
|  | **MS D** | 7.99±7.11 | 12.37±10.59 | 7.21±9.18 | 4.88 | 0.009 | 0.087 | -0.453 | 0.921 | 0.089 | 0.015 | 0.518 |
| Duration | **MS A** | 58.11±4.19 | 54.75±5.75 | 51.11±4.19 | 16.31 | <0.001 | 0.033 | 0.543 | <0.001 | 1.215 | <0.001 | 0.720 |
|  | **MS B** | 53.26±5.45 | 56.21±5.16 | 53.48±7.16 | 3.83 | 0.024 | 0.032 | -0.556 | 1 | -0.032 | 0.036 | 0.436 |
|  | **MS C** | 69.39±11.80 | 68.47±12.87 | 80.52±18.67 | 10.65 | <0.001 | 0.965 | 0.073 | 0.011 | -0.660 | <0.001 | -0.747 |
|  | **MS D** | 51.56±8.98 | 55.71±12.58 | 47.24±10.84 | 8.43 | <0.001 | 0.186 | -0.357 | 0.118 | 0.417 | <0.001 | 0.717 |
| Cover-age | **MS A** | 26.09±10.50 | 20.54±7.70 | 17.18±6.22 | 13.04 | <0.001 | 0.015 | 0.631 | <0.001 | 1.119 | 0.025 | 0.477 |
|  | **MS B** | 18.83±6.94 | 22.42±7.50 | 20.39±8.62 | 2.29 | 0.104 | - | - | - | - | - | - |
|  | **MS C** | 38.73±11.05 | 36.50±12.69 | 49.51±13.37 | 17.29 | <0.001 | 0.588 | 0.181 | <0.001 | -0.845 | <0.001 | -0.992 |
|  | **MS D** | 16.92±12.49 | 20.88±14.72 | 12.92±13.81 | 4.84 | 0.009 | 0.316 | -0.279 | 0.286 | 0.296 | 0.011 | 0.554 |
| Occurrence | **MS A** | 3.64 ± 0.88 | 3.14 ± 0.89 | 2.86 ± 0.77 | 8.40 | <0.001 | 0.031 | 0.558 | <0.001 | 0.947 | 0.130 | 0.325 |
|  | **MS B** | 3.01 ± 0.80 | 3.35 ± 0.79 | 3.17 ± 0.81 | 1.97 | 0.143 | - | - | - | - | - | - |
|  | **MS C** | 4.38 ± 0.49 | 4.18 ± 0.62 | 4.64 ± 0.51 | 10.45 | <0.001 | 0.203 | 0.338 | 0.040 | -0.518 | <0.001 | 0.806 |
|  | **MS D** | 2.57 ± 1.52 | 2.81 ± 1.44 | 1.94 ± 1.70 | 4.74 | 0.010 | 0.641 | -0.164 | 0.161 | 0.376 | 0.011 | 0.546 |

**S5. EEG source localization**

Figure S1. Estimated sources of the four microstates during mind-wandering. A distributed linear inverse solution (Loreta) using the MNI template brain was applied to each time point that was labelled with a given microstate, thresholded at >90% correlation. The sources were normalized by the mean activity over the whole EEG for each solution point. The source maps were then averaged across all time points for each microstate. The figure shows the average across the 150 participants.

**
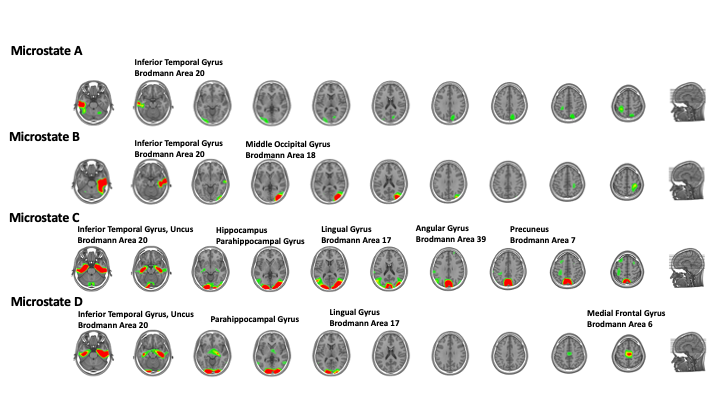
**

**S6. EEG microstates**

Figure S2. Group-level maps were estimated by separately clustering MCI patients, healthy older and healthy younger participants, and all subjects combined (All).


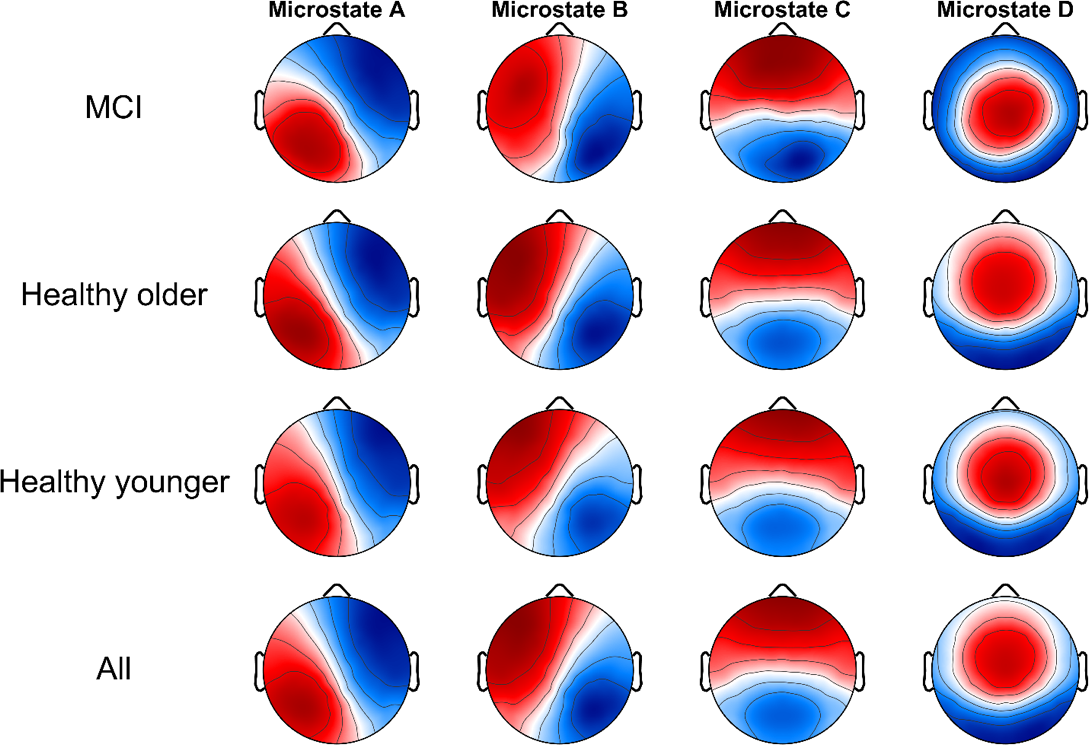


**S7. Neuropsychological Examination**

Table S4. Mean values and standard deviations for MCI patients.

| MCI patients | | | |
| --- | --- | --- | --- |
| Global Cognition | **Exams** | **Mean and ± SD** | **Score Range** |
|  | Mini-Mental State Exam (MMSE) | 26.4 ± 3.3 points | 0-30 points |
|  | Clock Drawing Test (CDT) | 8.8 ± 1.4 points | 0-10 points |
|  | Three objects-three places (3O3P) | 7.3 ± 2.2 points | 0-9 points |
|  | Montreal Cognitive Assessment Score (MoCA) | 23.4 ± 4.1 points | 0-30 points |
| Episodic memory | Immediate Selective Reminding Test | 14.2 ± 2.1 points | 0-16 points |
|  | Free Selective Reminding Test | 6.0 ± 2.6 points | 0-16 points |
|  | Cued Selective Reminding Test | 11.9 ± 3.9 points | 0-16 points |
|  | Rey–Osterrieth Complex Figure (ROCF) copy | 33.8 ± 1.9 points | 0-36 points |
|  | Rey–Osterrieth Complex Figure (ROCF) recall | 12.4 ± 7.7 points | 0-36 points |
|  | Logical Memory Story B (MEM-IV, immediate) | 10.1 ± 4.3 points | 0-25 points |
|  | Logical Memory Story B (MEM-IV, delayed) | 9.2 ± 4.8 points | 0-25 points |
|  | Memory Index Score (MoCA-MIS) | 9.0 ± 4.1 points | 0-15 points |
| Working Memory | Digit Span (WAIS-IV) | 8.2 ± 1.7 points | 0-16 points |
| Language | Semantic Fluency (fruits) | 14.2 ± 4.5 points | 120 sec |
|  | Phonemic Fluency (V) | 16.5 ± 5.9 points | 120 sec |
| Attention | Coding (WAIS-IV) | 48.6 ± 13.2 points | 0-135 points |
| Executive functioning | Trail-Making Test A (TMT-A) | 46.1 ± 16.5 sec | 0-300 sec |
|  | Trail-Making Test B (TMT-B) | 115.1 ± 64 sec | 0-300 sec |
| Depression and Anxiety | Anxiety Scale (HADS-A) | 5.6 ± 3.4 points | 0-21 points |
|  | Depression Scale (HADS-D) | 3.4 ± 3.1 points | 0-21 points |
